# Genome-wide identification and integrative analysis of KNOX family characterization, duplication and expression provide insights into PEG-induced drought stress in *Toona fargesii*

**DOI:** 10.1186/s12864-025-11628-4

**Published:** 2025-04-29

**Authors:** Qiuwei Zhong, Qiangqiang Cheng, Xuanjin Du, Yao Xiao, Chunce Guo, Lu Zhang, Jikai Ma

**Affiliations:** 1 Jiangxi Provincial Key Laboratory of Subtropical Forest Resources Cultivation, 2011 Co-Innovation Center of Jiangxi Typical Trees Cultivation and Utilization, Jiangxi Agricultural University, Nanchang, 330045 China; 2 Jiangxi Provincial Key Laboratory of Improved Variety Breeding and Efficient Utilization of Native Tree Species, Jiangxi Agricultural University, Nanchang, 330045 China; 3 Jiangxi Province Key Laboratory of Vegetable Cultivation and Utilization, Jiangxi Agricultural University, Nanchang, 330045 China

**Keywords:** *Toona fargesii*, KNOX, Gene family, Drought stress, Whole-genome duplication

## Abstract

*Toona fargesii* A. Chev. (*T. fargesii*), a precious tree with timber and medicinal properties, belongs to the *Toona* genus of the Meliaceae family. It is an endangered species in China, owing to various issues including the concerns about the drought aspect. KNOXs (knotted-like homeoboxes), a subset of TALE transcription factors, play pivotal roles in development and abiotic stress including drought resistance. The recent publication of the *T. fargesii* genome, indicating a specific whole-genome duplication (WGD) event in the *Toona* genus, serves as a valuable resource for uncovering the role of *KNOX* genes in *T. fargesii*. Here, genome-wide analysis including identification, synteny and duplication of *KNOX* genes was conducted to unveil their characterization and evolution. Moreover, gene structures, protein-protein interaction (PPI), subcellular localizations and expression patterns were also examined to verify *KNOX* genes with respect to drought response and development in *T. fargesii*. Generally, 21 putative *TfKNAT* (orthologs of KNAT) genes were identified and classified into three subfamilies. Intriguingly, most of *TfKNAT* gene possessed a paralog on another chromosome exhibiting high collinearity and similarities in chromosome regional assignments, sequences, structures, *cis*-elements, subcellular localizations and expression patterns. They diverged approximately 4.2 to 8.4 million years ago (MYA) approaching to the specific WGD (22.1 ~ 50.1 MYA) which may predominantly drive the family expansion. More importantly, the *cis*-elements contained many ABA-responsive elements strongly associated with drought stress, especially three *TfKNAT3/4* genes, and PPI analysis suggested that TfKNAT3/4 could interact with proteins related to the drought. Indeed, the expression of three *TfKNAT3/4* members sharply increased and then gradually decreased with prolonged PEG stress duration. Additionally, the ABA treatment significantly induced three *TfKNAT3/4* genes expression also strengthened their involvement in the drought stress. Collectively, our findings highlight the significance of the TfKNAT family and the potential role of *TfKNAT3/4* in drought resistance of *T. fargesii.*

**Supplementary Information:**

The online version contains supplementary material available at 10.1186/s12864-025-11628-4.

## Introduction

*Toona fargesii* (2n = 2x = 56) is a versatile tree species with pinnately compound leaves in the *Toona* genus of the Meliaceae family. This tree is used in folk medicine across many regions of southern Asia. The bark and roots of *T. fargesii* contain limonoids, which are essential medicinal components for treating parasites and rashes [1]. This species has often been misidentified as *Toona ciliata* M. Roem. (*T. ciliata*), as their overlapping distributions and similar morphologies, such as tall trees, compound leaves and reddish wood. *T. fargesii* is often called ‘Chinese mahogany’ thanks to its exquisite wood, which is utilized for high-grade furniture and decorations. Furthermore, *T. fargesii* is an endangered species in China due to anthropogenic factors and natural obstacles. It is widely distributed in the southeast of subtropical Asia and extensively cultivated in southern China, including Jiangxi, Hunan, Guangdong, Fujian, and Zhejiang provinces. However, the population of *T. fargesii* is sporadically distributed in small clusters, often approaching to water sources in the wild. Indeed, this species largely demands for water in afforestation. Notably, a recent study suggested that the growth of *T. ciliata*, a congeneric species of *T. fargesii*, is likely to continue to decline under drying conditions induced by global warming [2]. Hence, drought stress induced by climate change could be a threat that needs concerns in the conservation and breeding process of *T. fargesii*.

KNOXs are plant-specific TALE transcription factors that widely exist in the plant kingdom and are involved in plant growth, development and biological processes, such as leaf development, vascular cambium maintenance and meristem establishment [3–7]. Ever since Sarah Hake and her colleagues discovered a ‘*knotted-1*’ mutant with knotted nodules on leaves in maize, the *KNOX* genes have been identified in several species [8–10]. Notably, it is reported that several *KNOX* genes contribute to drought resistance [11–13]. For instance, *PagKNAT2/6b* can directly inhibit the synthesis of gibberellin (GA), altering plant architecture and improving drought resistance in Populus (*Populus alba* × *P. glandulosa*). Recently, *GhKNOX4-A* and *GhKNOX22-D*, homologs to AT5G11060.1 (*KNAT4*) and AT5G25220.1 (*KNAT3*), were identified in cotton (*Gossypium hirsutum*) as responsive to drought stress. These genes were suggested to enhance drought resistance through the interaction between *GhKNOX4-A* and *GhABF2*, which regulates ABA hormone levels to coordinate stomatal opening and manage oxidative stress [13].

KNOX proteins typically possess two helical structures (helix 1 and helix 2) with three additional amino acids, forming a homeodomain (HD) structure [14]. The HD domain at the C-terminus of KNOX proteins plays essential roles in functional mediation and interacts with specific sites on target genes through helical structures. In addition to the HD domain at the C-terminus, the N-terminus of KNOX contains a conserved MEINOX (MEIS-KNOX) domain composed of KNOX1 and KNOX2 domains, which recognize the promoters of target genes and exert specific regulatory effects. In addition, most KNOX proteins have evolved an ELK domain that may perform auxiliary functions [7]. Moreover, KNOX proteins can target genes that are critical for hormone mediation through conserved KNOX domains that orchestrate regulatory networks in plants [15, 16]. For example, a few KNOX genes target *GA20ox* genes, crucial gibberellin biosynthesis genes, through the recognition of sequences of *GA20ox cis*-regulatory elements, such as ‘TGAC’ and ‘GTGAC’, thereby degrading gibberellin levels [12, 15–19]. Additionally, both *OsIPT2* (*adenosine phosphate isopentenyltransferase 2*) and *OsIPT3*, which catalyze the rate-limiting step of CK biosynthesis, contain the binding motif ‘TGTGAC’ and can also be targeted by OSH15, a typical KNOX member to maintain cytokinin levels in rice [18, 20, 21].

*KNOX* genes are generally organized into three subfamilies, namely, class I *KNOX*, class II *KNOX* and class M *KNOX*, according to their sequence similarity, gene structure and gene expression pattern. In Arabidopsis, class I *KNOX* genes include *KNAT1/BP*, *KNAT2*, *KNAT6*, and *STM*, and class II *KNOX* genes are composed of *KNAT3*, *KNAT4*, *KNAT5*, and *KNAT7* [8, 22]. In addition, class M KNOX proteins are classified into a subgroup that lacks the ELK and HD domains [23]. The members of different subfamilies predominantly present distinct functions in plants. Early studies revealed that *KNOX* genes strongly contribute to leaf complexity [24–26]. For instance, a previous study revealed the important roles of KNOX genes in compound leaves during evolution in *Cardamine hirsuta*, a close relative of Arabidopsis with dissected leaves [24, 27]. In particular, class I *KNOX* genes, which are most closely related to leaf complexity and asymmetric development, have been extensively studied. *KNOX* genes play negative or positive roles in secondary cell wall deposition, which is critical for wood quality [10, 28, 29]. Especially, a few members of the class I subfamily have been identified as repressors that participate in secondary cell wall (SCW) formation [10, 29, 30]. KNOX1-overexpressing mutant plants present a reduction in lignin content in both maize and tobacco [31]. Similarly, a recent study reported that *PagKNAT2/6b*, an ortholog of *KNAT2* and *KNAT6*, was highly expressed in phloem and xylem and that overexpressing *PagKNAT2/6b* resulted in altered vascular patterns characterized by decreased secondary xylem with thin cell walls containing less cellulose, xylose and lignin in poplar (*P. alba × P. glandulosa*) [29]. In contrast to class I *KNOX* genes, class II *KNOX* genes exhibit widespread expression patterns underlying their multiple functions [32]. For example, *KNAT7*, a class II *KNOX* gene, also serves as a negative regulator of secondary wall biosynthesis and functions in a negative feedback loop that represses metabolically inappropriate commitment to secondary wall formation in Arabidopsis [33], whereas *KNAT7-1* regulates physical seed dormancy in mungbean (*Vigna radiata*) [34]. *KNOX4*, which is also a class II *KNOX*, controls seed physical dormancy in *Medicago truncatula* [35]. In *M. truncatula*,* MtKNOX4* plays a crucial role in integrating the CK pathway and boundary regulators, providing new insights into the roles of class II *KNOX* in regulating the elaboration of compound leaves [36]. In apple (*Malus domestica* Borkh.), *MdKNOX19*, a homolog of *KNAT3/4*, cooperate with *MdABI5* coordinating ABA levels [37].

Albeit *KNOX* has been characterized in Arabidopsis, Populus, and other species, little information is available regarding the *KNOX* in *T. fargesii*. Recently, the genomic study of *T. fargesii* identified a specific whole-genome duplication (WGD) event, offering valuable resources for investigating the role of *KNOX* genes in this species [38]. To primarily understand the changes of KNOX family genes during the evolution, genome-wide analysis was performed to elucidate the identification, classification, evolution, structure of the TfKNAT family. More importantly, the characterization, sequence similarity, expression patterns, regulatory networks and subcellular localization of *TfKNAT* genes were conducted to deeply explore their potential value contributing to the drought and development such as wood formation in *T. fargesii*. This study may lay foundation for the identification, characterization, expression pattern of TfKNAT family and provide insights into and evolution of gene families in the *Toona* genus.

## Methods and methods

### Plant materials and genetic information

The materials, including roots, leaves (leaflets and leaf petioles), flowers, buds and stems (xylem and phloem tissues), were sampled from mature trees of *T. fargesii* cultivated at Jiangxi Agricultural University, Nanchang city, China (E115°49’36”, N28°45’45”). The samples were stored in a -80 °C refrigerator to prepare for gene cloning and qRT‒PCR (quantitative real-time PCR) verification. The genome of *T. fargesii* used for analysis and set to be released on October 18, 2025, was obtained from our group [38]. The genomic information of other species, including *A. thaliana*, was downloaded from the phytozome database (https://phytozome-next.jgi.doe.gov/). Moreover, the tobacco plants (*Nicotiana benthamiana*) used for the subcellular location assay were cultivated under long-day conditions (illumination at 23 °C for 16 h and darkness at 16 °C for 8 h) and then transplanted to pots filled with a mixture of soil, vermiculite or sand at a 1:1:1 ratio.

### Identification and physicochemical properties of KNOX family in *T. fargesii*

Since KNOX typically contains four conserved domains: PF05920 (homeobox KN domain), PF03790 (KNOX1 domain), PF03791 (KNOX2 domain), and PF03789 (ELK domain), these domains were obtained from the Pfam database according to a previously reported KNOX family [39]. Then, redundant sequences were manually removed. To further examine potential members of the KNOX family, a hidden Markov model (HMM) was performed by using HMMER (v3.0) with Pfam sequences. The sequences with homologous alignment and HMM (e value < 1e-20) were considered candidate *KNOX* genes. The candidate sequences were further screened by the KNOX domains via PFAM (http://pfam.xfam.org/) and SMART (http://smart.embl-heidelberg.de). The Basic Local Alignment Search Tool (BLAST v2.14) was also employed to generate homologous sequences between the KNAT and TfKNAT proteins [40].

To predict KNOX properties in *T. fargesii*, the physicochemical properties of each putative TfKNAT protein were analyzed via ExPASy (https://www.ExPASy.org). SWISS-MODEL (swissmodel.expasy.org) and I-TASSER (https://zhanggroup.org/I-TASSER) were employed to predict the three-dimensional structure of the TfKNOX proteins. The subcellular localization of TfKNAT proteins was anticipated by using Cell-Ploc with the Plant-mPLoc model (http://www.csbio.sjtu.edu.cn/bioinf/plant-multi).

### Phylogenetic tree, chromosome localization and collinearity analysis

Clustal Omega (https://www.ebi.ac.uk/services) was used for sequence analysis of *KNOX* members from seven species, including *T. fargesii*,* Arabidopsis thaliana*, *Zea mays*, *Oryza sativa*, *Populus trichocarpa*, *Solanum lycopersicum*, and *Allium sativum*, which were downloaded from PlantTFDB v5.0 (https://planttfdb.gao-lab.org/index.php). KNOX members of these species were aligned via MAFFT (v7.0) with default parameters. MEGA (v11.0) was employed to analyze these KNOX sequences and construct a phylogenetic tree by the Maximum Likelihood (ML) method with 1,000 bootstrap repeats. Evolview v2.0 (http://www.evolgenius.info/evolview) was subsequently used to polish the figure of the phylogenetic tree. To better understand gene evolution and duplications, we extracted the chromosome distribution of *TfKNAT* members from the annotation of the *T. fargesii* genome. Then, collinearity analysis was conducted to further verify the gene duplications within the species. In addition, we performed collinearity analysis of the *KNOX* genes among *T. fargesii*, *T. sinensis* (A.Juss.) M.Roem. (*T. sinensis*) and *A. thaliana*.

Ka/Ks (nonsynonymous substitution rate/synonymous substitution rate) ratios were calculated to examine the selection pressure on the evolution of *TfKNAT* genes calculated by using MCScanX [41]. The ratio of 𝐾𝑎/𝐾𝑠 above 1.0 indicates purifying selection, whereas the ratio of 𝐾𝑎/𝐾𝑠 below 1.0 suggests purifying or negative selection [43]. Besides, the divergence time was calculated by the formula *T* = *Ks*/2*r*, with *Ks* being the synonymous substitutions per site and *r* being the rate of divergence for nuclear genes from plants. The *r* was taken to be 1.5 × 10^− 8^ synonymous substitutions per site per year for dicotyledonous plants [42, 43]. Tandem duplication referred to duplicates that were next to each other in the genome, leading to rapidly expanding gene families. Segmental duplication involved larger sections of the genome being duplicated, potentially resulting in greater functional diversity. Tandem duplication was identified by duplicates that were next to each other in the genome and were less than 10 kb in length. Additionally, duplicates that were longer than 10 kb were regarded as segmental duplications [44, 45].

### Gene structure and protein motif, *cis*-elements and protein–protein interaction (PPI) analysis

The conserved motifs of the TfKNAT protein were identified by the MEME suite (http://memesuite.org) [46]. After that, NCBI Batch Web CD-Search (https://www.ncbi.nlm.nih.gov/Structure/bwrpsb/bwrpsb.cgi) was carried out to predict the conserved domains of the TfKNAT protein family in *T. fargesii*. Additionally, pygff (v2.0) was used to extract the 2000 bp upstream sequences of the *TfKNAT* genes as promoter regions. The PlantCARE (http://bioinformatics.psb.ugent.be/webtools/plantcare/html) database was used to indentify the *cis*-elements in the extracted sequences. Besides, we manually optimized the *cis*-elements and supplemented several drought related *cis*-regulatory elements including ABA-responsive element, dehydration responsive element, antioxidant responsive element, W-box and TCA-element. Finally, the figures of the motifs, gene structures, *cis*-elements and responsive functions were merged and visualized.

The homologous gene sequences of Arabidopsis were input into the STRING (https://cn.string-db.org/) database to construct a protein‒protein interaction (PPI) network of TfKNAT proteins (https://string-db.org/) [47]. The homologous protein sequences were subsequently aligned against the *T. fargesii* genome. The genes of Arabidopsis were substituted with homologous genes of *T. fargesii* [48]. The PPI networks were established by Cytoscape v3.7.2 (https://cytoscape.org/).

### The isolation of *TfKNAT* genes and vector construction for subcellular localization

Total RNA was extracted from different tissues of *T. fargesii* from the materials described according to the instructions of the Total RNA Extraction Kit for Plants (provided by TianGen Biotech). The concentration, purity, and quality of the RNA were detected using a nucleic acid spectrophotometer and 1.0% agarose gel electrophoresis. Qualified RNA samples were then reverse transcribed into cDNA using the EasyScript^®^ First-Strand cDNA Synthesis SuperMix Reverse Transcription Kit (provided by QuanSheng Biotech Co., Ltd.) and stored at -20 °C. Next, specific primers (Table S2) were designed via Oligo (v7.0) software and synthesized by Sangon Biotech (Shanghai) Co., Ltd. The PCR amplification system consisted of a 50 µL reaction mixture, following the instructions of the 2×TransStart^®^FastPfu Fly PCR SuperMix, which contained 1 µL of cDNA template, 25 µL of 2×TransStart^®^FastPfu Fly PCR SuperMix, 1 µL of each of the forward and reverse primers (10 µmol/L), and 18 µL of nuclease-free water. The PCR amplification program for the target gene reaction was as follows: initial denaturation at 98 °C for 1 min; denaturation at 98 °C for 10 s, annealing at 58 °C for 5 s, and extension at 72 °C for 10 s for 30 cycles; and a final extension at 72 °C for 1 min. The PCR products were preliminarily detected for size and quality using 1.5% agarose gel electrophoresis.

To better know the characterization of *TfKNAT* genes, the subcellular localization experiment was conducted. The primers were designed using Oligo (v7.0) to amplify and purify the *TfKNAT* family genes (Table S2). The pCAMBIA1304 vector containing the 35S promoter and GFP was digested with the *Nco*I and *Spe*I restriction endonucleases. The amplified gene and the digested vector were ligated to construct a vector, pCAMBIA1304-*TfKNAT*-GFP, driven by the 35S promoter. The ligated plasmid was subsequently transformed into competent *Agrobacterium tumefaciens* strain GV3101 cells, which were cultured on LB supplemented with kanamycin at 28 °C for 2‒3 days. Single colonies were picked, and the positive strains were screened via PCR and then verified via first-generation sequencing (Shanghai Biotech Co., Ltd.). After the construction of the overexpression vector, the pCAMBIA1304-GFP and pCAMBIA1304-TfKNAT-GFP fusion vector strains were infiltrated into five-week-old *N. benthamiana* leaves via a buffer mixture (10 mM MES pH 5.6, 10 mM MgCl_2_, or 200 µM acetosyringone) [49]. After 24 h of incubation in the dark, the GFP signals were detected using a confocal laser scanning microscope (OLYMPUS FV3000).

### Drought stress and hormones treatments and expression patterns determined by qRT‒PCR

As mentioned above, warming and drought conditions might threaten *T. fargesii*, and *KNOX* genes significantly contribute to the drought resistance of many plants. One-year-old *T. fargesii* seedlings were used for the drought stress experiment. The drought stress experiments were designed including a PEG treatment at a concentration of 75 mM to simulate drought conditions according to previous studies [50, 51]. The treatment durations were 0 h, 6 h, 12 h, and 24 h, respectively. Five plants from each treatment were used as replicates. Furthermore, previous studies have shown that KNOX is closely related to the regulation of plant hormones. To investigate the hormones impact on gene expression, we applied treatments with three hormones: cytokinin (kinetin, 10 µM), auxin (IAA,10 µM), and abscisic acid (ABA, 100 µM), which is highly associated with the drought stress. The seedlings were sprayed with these hormones, and samples were collected 6 h later to assess relative expression levels of KNOX family members according to the PEG treatment results.

The primers for qRT‒PCR were designed by using Oligo v7.0 to analyze the expression pattern of each TfKNAT family member. To specify primer sequences and avoid interference from homologous sequences, the primers were aligned against the genome (Table S1). The *actin* gene was used as the internal reference gene [52]. The PCR conditions were as follows: 60 s at 95 °C for initial denaturation, followed by 40 cycles of 15 s at 95 °C, 60 s at 60 °C, 15 s at 95 °C, 30 s at 60 °C, and 15 s at 95 °C for annealing. The standard deviations (SDs) were calculated in triplicate. The relative expression levels were calculated using the 2^−ΔΔCT^ method. The statistical analysis of the data was performed by SPSS (v24.0) with one-way ANOVA model and LSD test.

## Results

### Identification of *TfKNAT* genes and analysis of their physicochemical properties

**Fig. 1:**
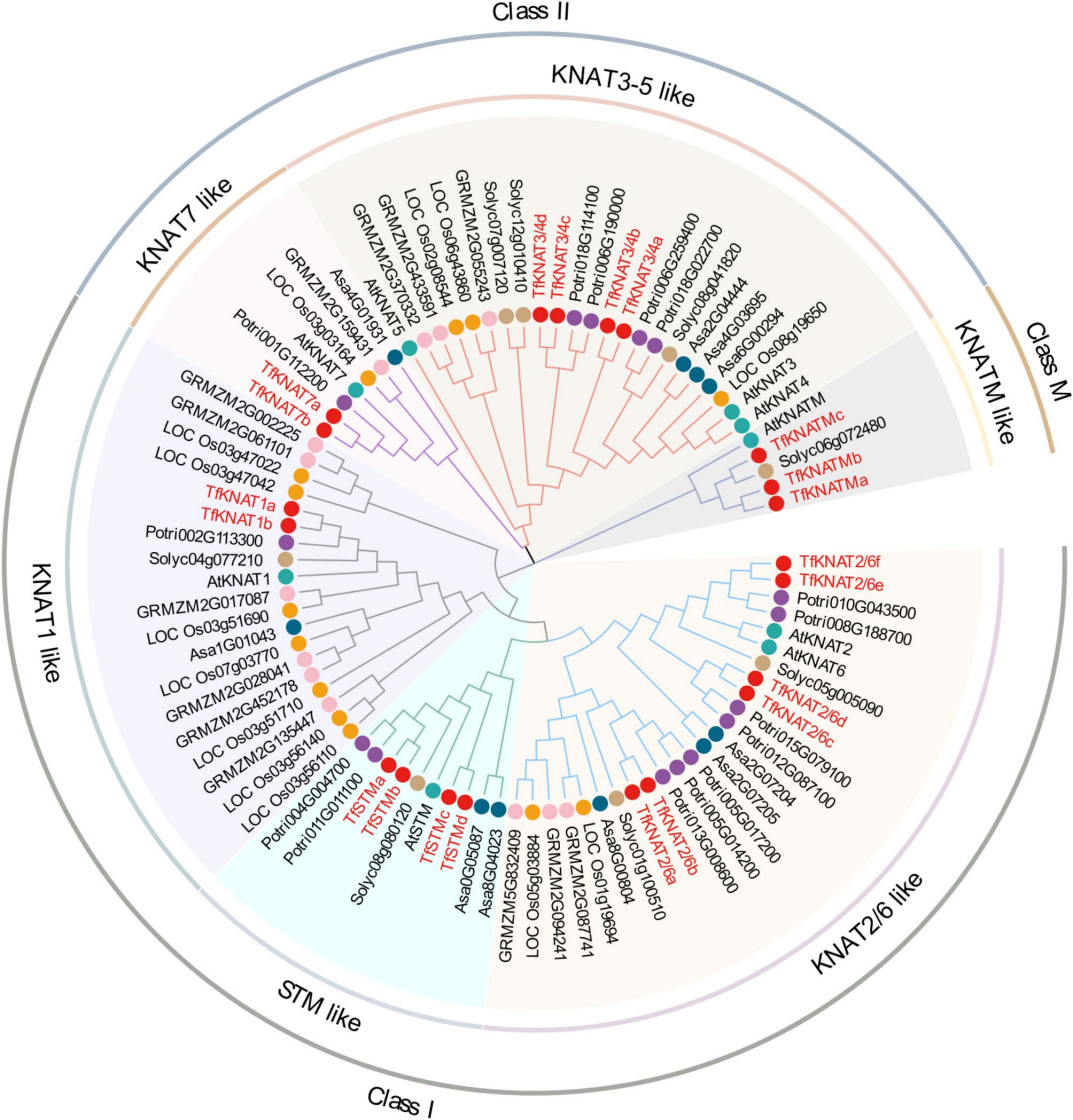
Phylogenetic tree analysis of KNOX members among *T. fargesii*, *A. thaliana*, *Z. mays*, *O. sativa*, *P. trichocarpa*, *S. lycopersicum*, and *A. sativum* species. The red names indicate the members of TfKNAT of *T. fargesii*

To better extract the members of KNOX in *T. fargesii*, HMMER and BLAST were both implemented, and their results were unified. To reveal the evolutionary relationships of the *TfKNAT* genes, a phylogenetic tree was constructed using the KNOX protein sequences derived from six species, including *A. thaliana*, *Z. mays*, *O. sativa*, *P. trichocarpa*, *S. lycopersicum*, and *A. sativum* (Fig. 1; Table S3). Consequently, a total of 21 *TfKNAT* members were identified and clustered into three subfamilies composed of six subclades. TfKNATs are classified into three subfamilies: class I KNOX, class II KNOX and class M KNOX. The class I KNOX subfamily was divided into three clades, namely, KNAT1-like, STM-like, and KNAT2/6-like; class II had two clades, namely, KNAT7-like and KNAT3/4-like; and class M possessed only one clade, namely, KNATM-like, which included TfKNATMa, TfKNATMb and TfKNATMc three members, according to the classification of *KNOX* genes in Arabidopsis (Fig. 1).

Furthermore, the characterization of proteins encoded by *TfKNAT* genes was presumed by multiple methods. First, the length of the protein encoded by the *TfKNAT* genes varied from 134 aa to 521 amino acid (aa). The shortest amino acid sequence was TfKNATMa, which is 134 aa in length, whereas the longest sequence was encoded by the *TfKNAT3/4a* gene, which is 521 aa in length. Additionally, the relative molecular weights of the proteins encoded by the *TfKNAT* genes ranged from 15531.21 to 58958.71 Daltons (Da) and were positively correlated with amino acid length. Moreover, the theoretical isoelectric point analysis exhibited that all of the KNOX proteins exhibit isoelectric points below 7.0, indicating that they both encode acidic amino acid sequences. Among them, TfKNAT2/6b presented the lowest isoelectric point at 4.82, while TfKNAT3/4a presented the highest isoelectric point (pI) at 6.68. In addition, the instability coefficients ranged from 40.29 to 64.73, which were greater than 40.0, indicating that all the TfKNAT proteins were unstable and that all the TfKNAT proteins were hydrophilic, and the prediction of their subcellular localization revealed that they were predicted to be located in the cell nucleus (Table 1).

**Table 1:** Basic information of the TfKNAT in *T. fargesii*

Gene name	Number of amino acid (aa)	Molecular weight (Da)	Theoretical pI	Instability index	Aliphatic index	Grand average of hydropathicity	Subcellular localization prediction
*TfKNAT1a*	365	41525.39	6.09	49.03	61.26	-0.899	Nucleus.
*TfKNAT1b*	365	41644.42	6.1	50.17	59.64	-0.924	Nucleus.
*TfKNAT2/6a*	330	37558.01	4.93	49.43	62.15	-0.762	Nucleus.
*TfKNAT2/6b*	336	38379.77	4.82	47.17	61.9	-0.757	Nucleus.
*TfKNAT2/6c*	319	35954.33	5.24	34.34	68.24	-0.636	Nucleus.
*TfKNAT2/6d*	318	35818.22	5.22	34.37	69.62	-0.567	Nucleus.
*TfKNAT2/6e*	309	35415.02	4.84	40.33	70.78	-0.644	Nucleus.
*TfKNAT2/6f*	313	35764.46	5.09	40.11	72.68	-0.592	Nucleus.
*TfKNAT3/4a*	521	58958.71	6.68	49.63	76.05	-0.772	Nucleus.
*TfKNAT3/4b*	446	50396.49	5.71	51.17	70.85	-0.854	Nucleus.
*TfKNAT3/4c*	353	39927.36	5.66	55.34	68.53	-0.833	Nucleus.
*TfKNAT3/4d*	353	40018.44	5.6	50.65	69.07	-0.862	Nucleus.
*TfKNAT7a*	297	33582.92	6.32	59.18	84.38	-0.612	Nucleus.
*TfKNAT7b*	287	32739.97	6.32	64.73	83.59	-0.655	Nucleus.
*TfKNATMa*	134	15531.21	5.39	65.75	60.45	-1.073	Nucleus.
*TfKNATMb*	140	16099.76	5.22	63.13	62.79	-1.037	Nucleus.
*TfKNATMc*	162	18401.87	4.98	42.14	77.72	-0.663	Nucleus.
*TfSTMa*	301	34064.40	5.58	56.4	64.55	-0.662	Nucleus.
*TfSTMb*	359	40550.47	6.14	52.35	57.63	-0.736	Nucleus.
*TfSTMc*	327	36177.98	5.92	38.83	67.77	-0.488	Nucleus.
*TfSTMd*	327	36448.18	5.68	40.29	64.22	-0.58	Nucleus.

### Analysis of *TfKNAT* gene location, collinearity and duplication

**Fig. 2:**
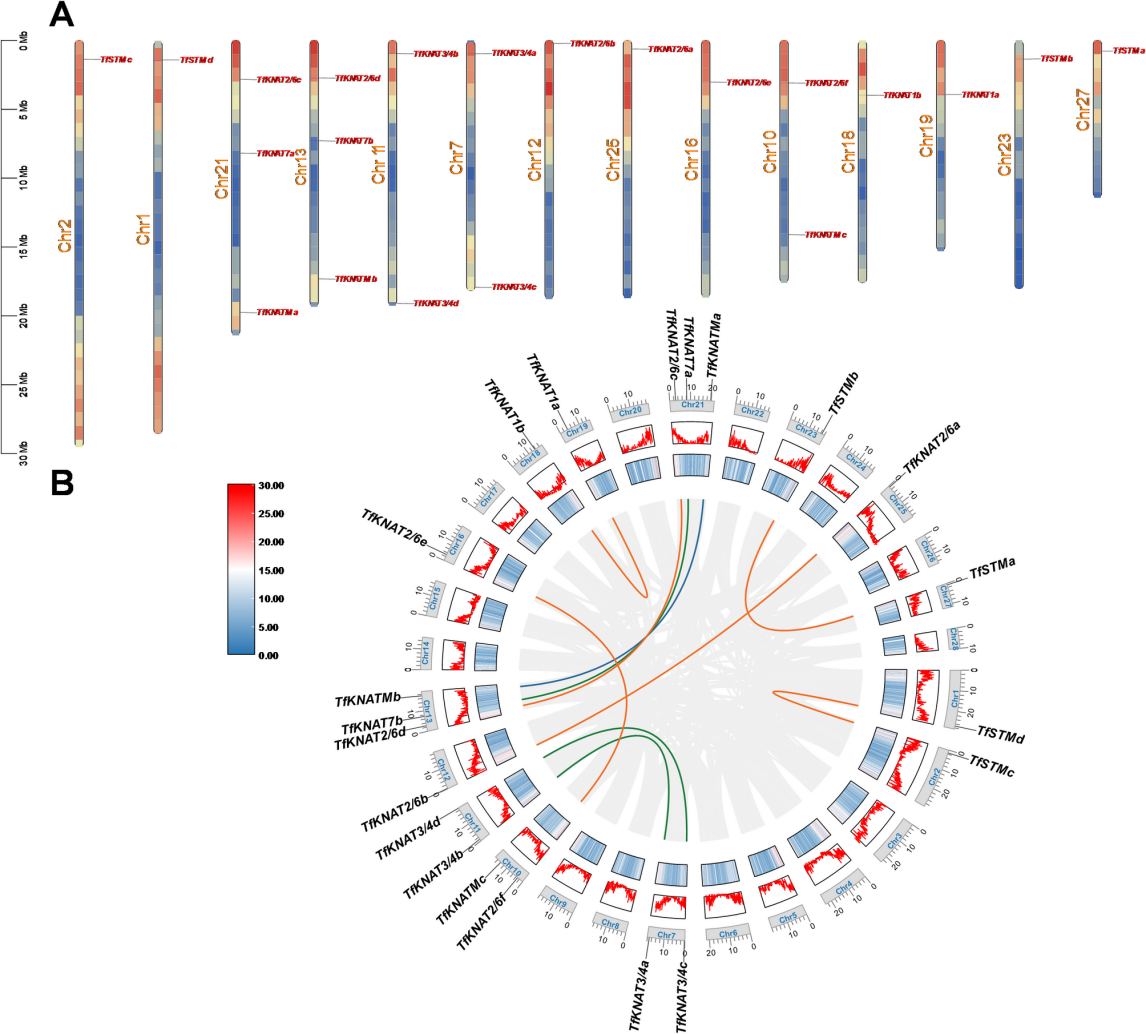
Gene locations and synteny of *TfKNAT* genes. (**A**) Gene location and duplication analysis of *TfKNAT* genes. Red signifies a high density of genes on the chromosome; blue represents a low density of genes on the chromosome. (**B**) Gene locations and interchromosomal relationships of *TfKNAT* genes. The circos plot represents, from inside to outside, synteny relationships between chromosomes, GC content, gene density and chromosomes. The orange lines represent pairs of syntenic relationships in the class I KNOX family, the green lines indicate pairs of syntenic relationships in the class II KNOX family, and the blue lines signify pairs of syntenic relationships in the class M KNOX family

To further understand the *TfKNAT* gene location on the chromosomes (Chr), the chromosomes were adjusted to be in same direction according to the gene density on the chromosome. Generally, all the *TfKNAT* genes were distributed on the 14 chromosomes, including Chr1, Chr7, Chr10, Chr12, Chr13, Chr16, Chr21, Chr19, Chr23, and Chr25 (Fig. 2A). Notably, the genes *TfKNAT2/6d*, *TfKNAT7b*, and *TfKNATMb* were sequentially colocalized on Chr13, resembling gene linkages, and their corresponding paralogs, *TfKNAT2/6c*, *TfKNAT7a*, and *TfKNATMa*, colocalized on Chr21 in the same order. Additionally, a similar phenomenon was observed on Chr7 and Chr11, where *TfKNAT3/4a* and *TfKNAT3/4c* were colocalized on Chr7, while their paralogs, *TfKNAT3/4b* and *TfKNAT3/4d*, were similarly colocalized on Chr11 (Fig. 2A).

To further examine the relationships among the *TfKNAT* genes within the species, interspecies collinearity analysis was systematically conducted. Here, the *TfKNAT* gene family exhibited a significant paralogous relationship, which might have originated from large-scale chromosome duplications, leading to *TfKNAT* gene family expansion. Except for Chr23 and Chr27, each paralogous pairs were separately located on two different chromosomes whose lengths and gene density were relatively similar. Moreover, these paralogs displayed high collinearity (Fig. 2B).

Additionally, the 𝐾𝑎/𝐾𝑠 values of the paralogous pairs were both less than 1.0, suggesting that they experienced purifying selection (Table S4). No tandem duplications were detected on any of the chromosomes. In addition to *TfKNATMa*, all the *TfKNAT* genes underwent duplications in the genome. Furthermore, we calculated the divergence time between the paralogs, and the divergence time ranged from 4.2 to 8.4 MYA. Interestingly, this time range was close to the WGD in the *Toona* genus (22.1 ~ 50.1 MYA). Strong collinearity was also detected between the chromosomes on which pairs of paralogs were separately located.

To better reveal the homologous relationship between different species, intraspecies synteny analysis was conducted among *T. fargesii* and two representative dicot plants, *A. thaliana* and *T. sinensis*, a close relative of *T. fargesii*, to further examine the relationships among the *KNOX* genes. In total, 21 pairs of homologs containing 65 synteny lines were found between *T. fargesii* and *T. sinensis*, indicating close relations of *KNOX* members between the species. Moreover, seven pairs of homologs with 29 syntenies were identified between *T. fargesii* and *A. thaliana* (Fig. S1). The *KNAT* genes in Arabidopsis corresponded to their homologous genes in *T. fargesii* partially support the reliability of *TfKNAT* identification. Besides, this result was correlated with the close relationships between *T. fargesii* and *T. sinensis* underlying homologous evolution in the *Toona* genus.

### Analysis of the relationships, gene structure and motifs of the TfKNAT family

**Fig. 3:**
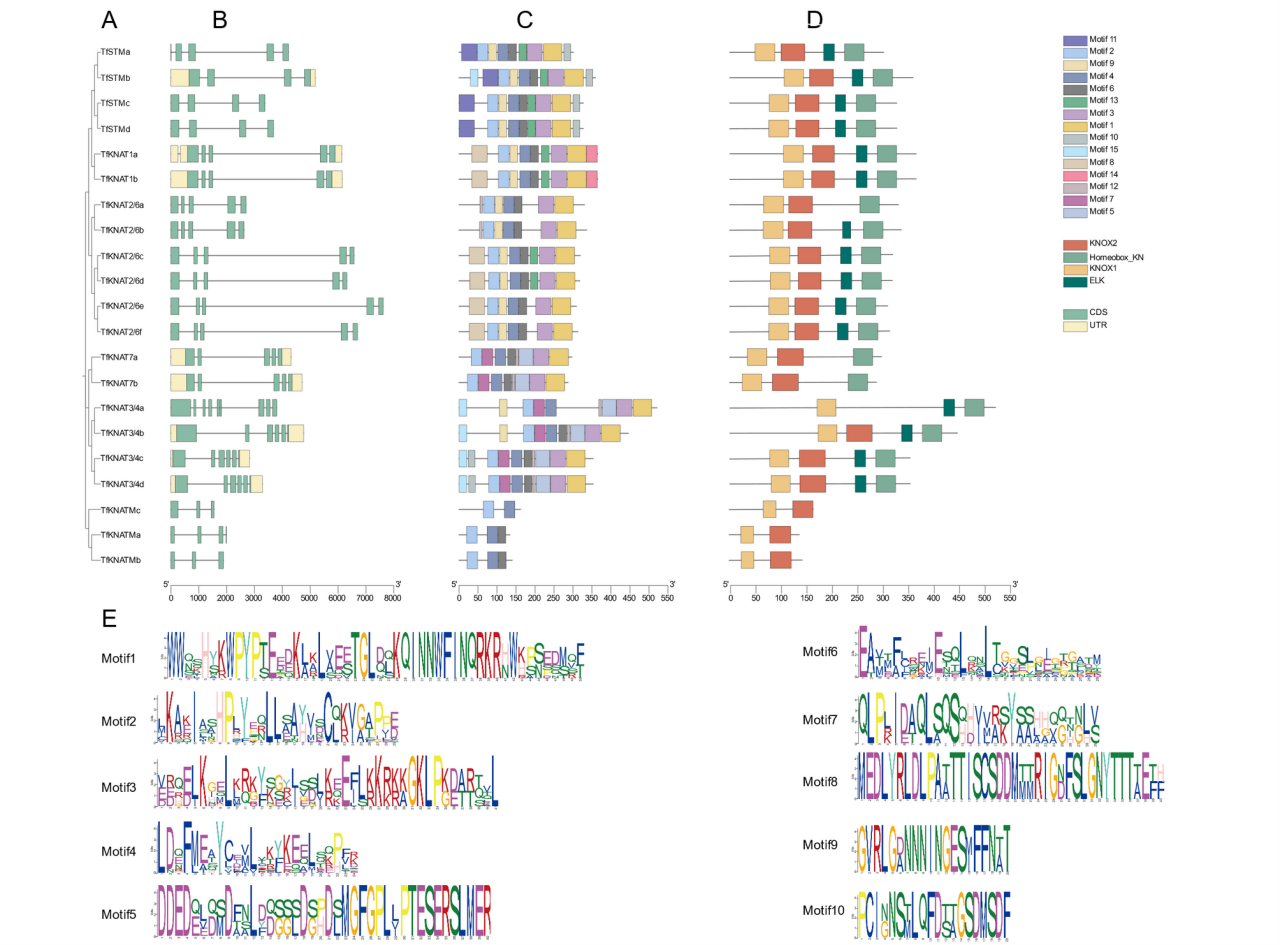
Phylogenetic tree, encoding regions, motifs and gene structure analysis of TfKNAT. (**A**) Relationships of TfKNAT family members; (**B**) coding structure of TfKNAT; (**C**) conserved motifs; (**D**) conserved domains; (**E**) sequences of the motifs

Interestingly, ten pairs of paralogs were identified in the family, and the sequence similarity of the paralog pairs was greater than 79%, except for *TfKNAT3/4a* and *TfKNAT3/4b*, which had a similarity of 68.54% (Fig. 3A, Table S4). On the basis of the gene structure information extracted from the genome, analysis of the gene structures displayed that the *TfKNAT* genes presented similar structures, including UTRs, CDSs, and introns, between the paralogs. For instance, the *TfKNAT2/6c* and *TfKNAT2/6d* presented 90% sequence similarity and displayed almost the same UTRs, CDSs, and introns (Fig. 3B). Remarkably, TfKNAT2/6a, TfKNAT7a, TfKNAT7b, TfKNATMa, TfKNATMb and TfKNATMc lost an ELK domain. Indeed, KNOX typically contains four conserved domains: the KNOX1 domain, the KNOX2 domain, the ELK domain, and the homeobox KN domain. Nevertheless, TfKNATMa and TfKNATMb only possessed two conserved domains, namely, the KNOX1 domain and the KNOX2 domain, indicating that they belong to the same subfamily, KNOX M, which was a family reported more recently (Fig. 3D). Furthermore, TfKNAT3 lacked the KNOX2 domain, and it also lacked a UTR at the 5’ end and a motif6 (Fig. 3C and E). Moreover, the conserved protein motifs of the *TfKNAT* genes were analyzed by using the MEME suite, and a total of fifteen conserved motifs were identified. The length of the motifs ranged from 20 to 50 amino acids. In general, the structure of *TfKNAT* genes varies slightly within the family corresponding to its versatile functions. Generally, most paralogs presented similar structures, including sequences, motifs, conserved domains and CDSs, indicating that the identification of *TfKNAT* genes was reliable and suggesting similar functions among the paralogs.

**Fig. 4:**
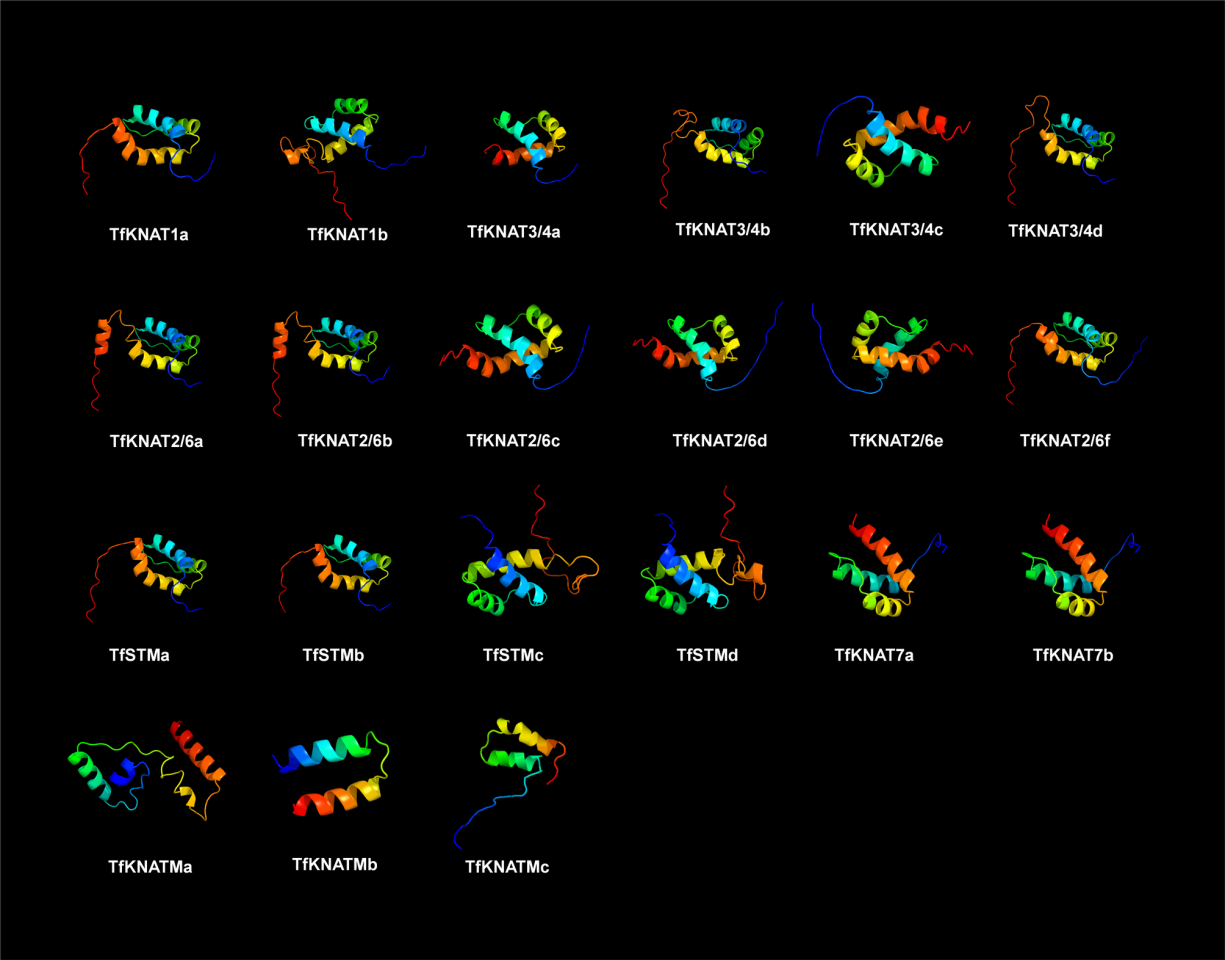
Three-dimensional structure of TfKNAT proteins

To further characterize the proteins, the three-dimensional structures of the TfKNAT proteins were predicted and visualized (Fig. 4). Except for the TfKNATM subclade, most TfKNAT proteins contained three typical three-helix structures that corresponded to three conserved domains, namely, the KNOX1, KNOX2, and homeobox KN domains. Additionally, except for the TfKNATM subclade, we found that the protein structures within the same subclade were highly similar, suggesting that they may have similar functions.

### The *cis*-elements in upstream regions of *TfKNAT* genes

**Fig. 5:**
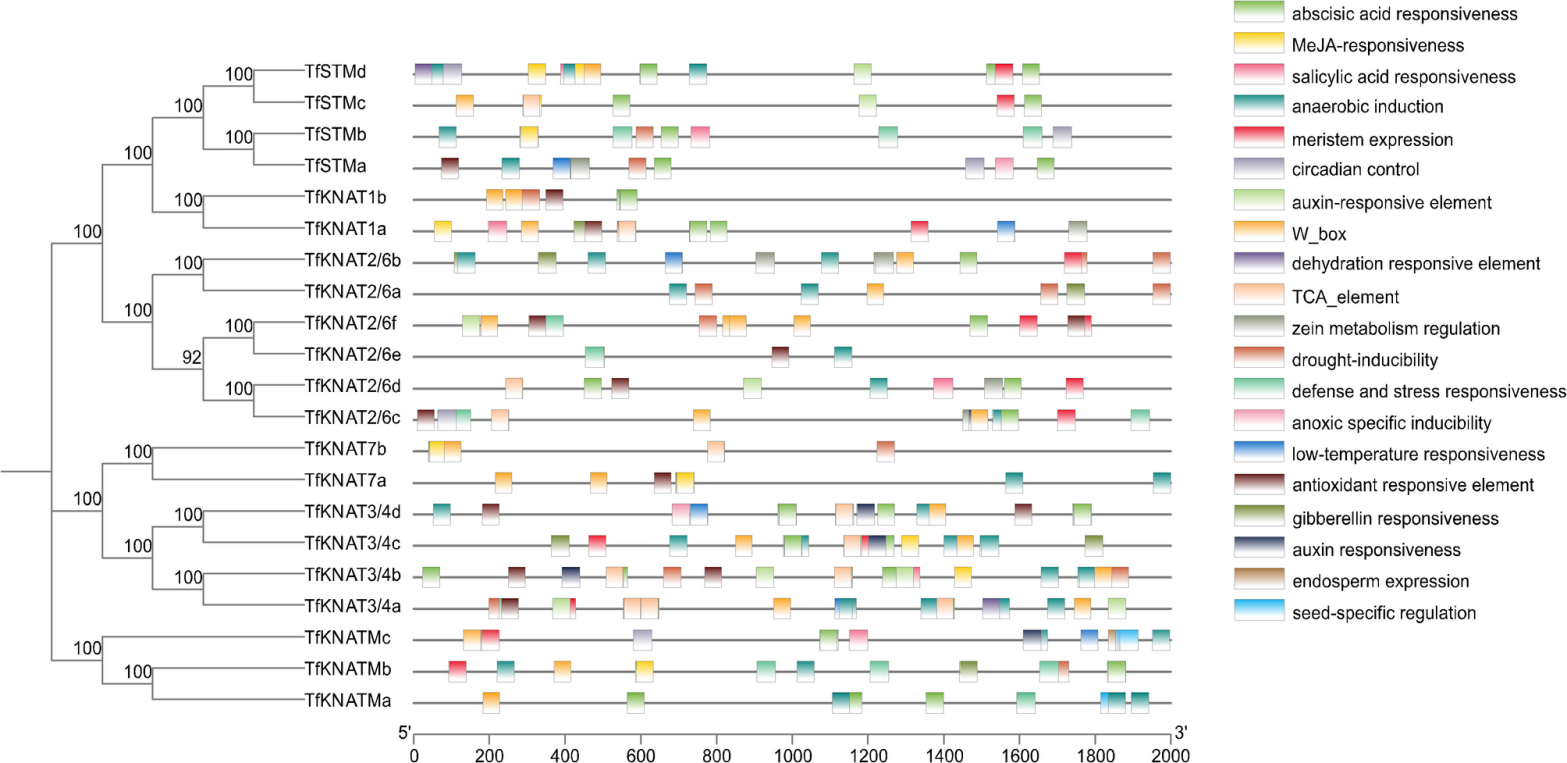
Analysis of *cis*-regulatory elements in TfKNAT members. The numbers on the branches signify the reliability of the relationship

To better understand the regulatory elements of *TfKNA*T genes, the *cis*-regulatory elements in the 2000 bp upstream promoter sequences of the TfKNAT coding regions were analyzed via PlantCARE to examine the potential regulation of *TfKNAT* genes. The core *cis*-elements in the promoter region of *TfKNAT* genes are diversely include lots of elements responsive to plant hormone signals and environmental stresses. Among them, many *cis*-elements are associated with hormones, such as auxin, abscisic acid (ABA), cytokinin and MeJA, suggesting regulatory interactions between *KNOX* and hormones (Fig. 5). In addition, a total of 20 types of *cis*-acting elements related to the regulation of plant physiological activities were examined. Even though the coding sequences displayed high similarity among the paralogs, a few pairs of paralogs slightly varied in *cis*-elements. Analysis of these hormone-related *cis*-elements suggested that all the genes upstream regions, except for *TfKNAT2b*, contained the ABA-responsive element (ABRE) and *TfKNAT3/4* subclade gene promoters had the most ABREs which are highly associated with drought stress (Fig. 5). Besides, the promoters of *TfKNAT3/4* contained the most elements related to the drought stress, including dehydration responsive element, antioxidant responsive element, W-box and TCA-elements. Of these, the promoter of *TfKNAT3/4d* possessed 13 G-box elements, indicating the presence of numerous binding sites for various transcription factors. Execpt for the *TfKNAT3/4a* promoter, which had only two ABREs, the other *TfKNAT3/4* promoters possessed more than six ABREs. The *TfKNAT3/4d* promoter had eight ABREs, indicating that the regulation of *TfKNAT3/4d* gene expression is closely related to ABA regulation (Fig. S2). Additionally, two auxin-responsive cis-acting elements, AuxRR-core and TGA-element, were identified in this study. Generally, most of the *cis*-elements gave rise to drought stress.

### Interplay of TfKNAT members

**Fig. 6:**
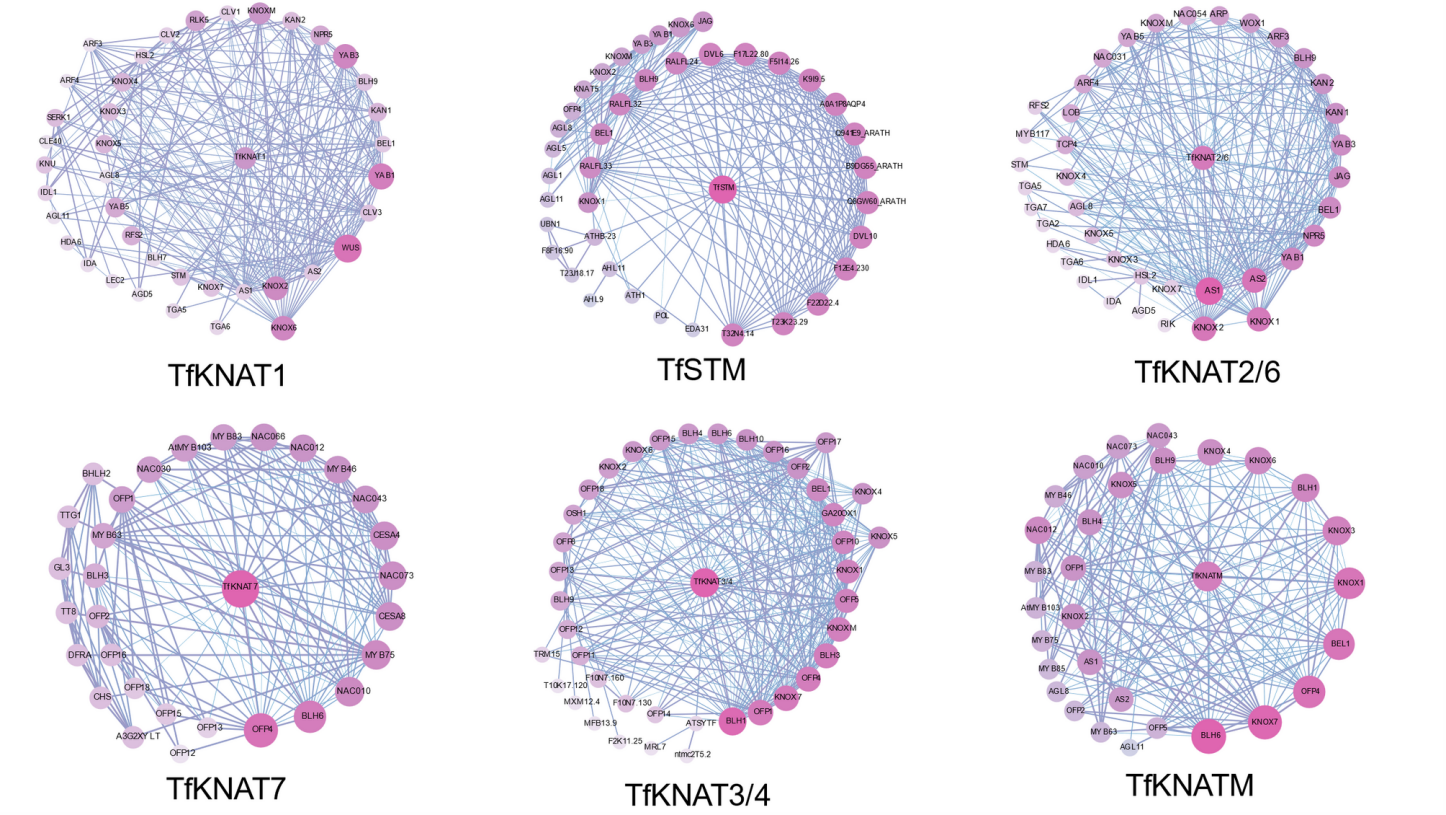
Protein‒protein interaction (PPI) networks of TfKNAT. The purple color and large circle represent strong interactions between the proteins. The dark purple color indicates strong interaction correlations

KNOX proteins are involved in large-scale plant development and interact with multiple genetic factors to orchestrate complex regulatory networks. As no studies have reported *KNOX* genes in *T. fargesii*, we referred to the homologs of KNOX proteins in Arabidopsis to better represent the putative regulatory networks (Fig. 6). First, interactions within the family were complex, suggesting KNOXs versatile functions in coordination. For example, TfKNAT1 was predicted to significantly interact with KNOX2 and KNOX6. Second, most of the regulators were transcription factors, such as NAC, BLH, MYB, WUS, AS1 and AS2. In particular, TfKNAT7 and TfKNATM interact with NACs, which are also vital transcription factors involved in biological regulation processes, especially abiotic stresses, indicating that these two members might participate in the abiotic resistance of *T. fargesii*. Third, as demonstrated above, regulators involved in the production of hormones, such as GA and auxin, were found in the networks. Specifically, GA20ox was associated with GA regulation, and ARF was related to auxin regulation, indicating its potential involvement in hormone regulation. TfKNAT3/4 can interact with BHL1, typically forming a dimer to regulate ABA hormones and increase drought resistance. TfKNAT7 can interact with OFP, which might be related to regulating secondary cell wall and lignin formation, such as OFP2 and OFP4 [53]. Additionally, the interaction between TfKNAT3/4 and KNOX7 also suggests that TfKNAT3/4 members may act as potential transcriptional activators, working together with KNAT7 to promote secondary cell wall biosynthesis [53, 54]. This finding supports our result that KNAT7 was expressed at lower levels in the xylem than in the phloem.

### Subcellular localization and gene interactions of TfKNAT

**Fig. 7:**
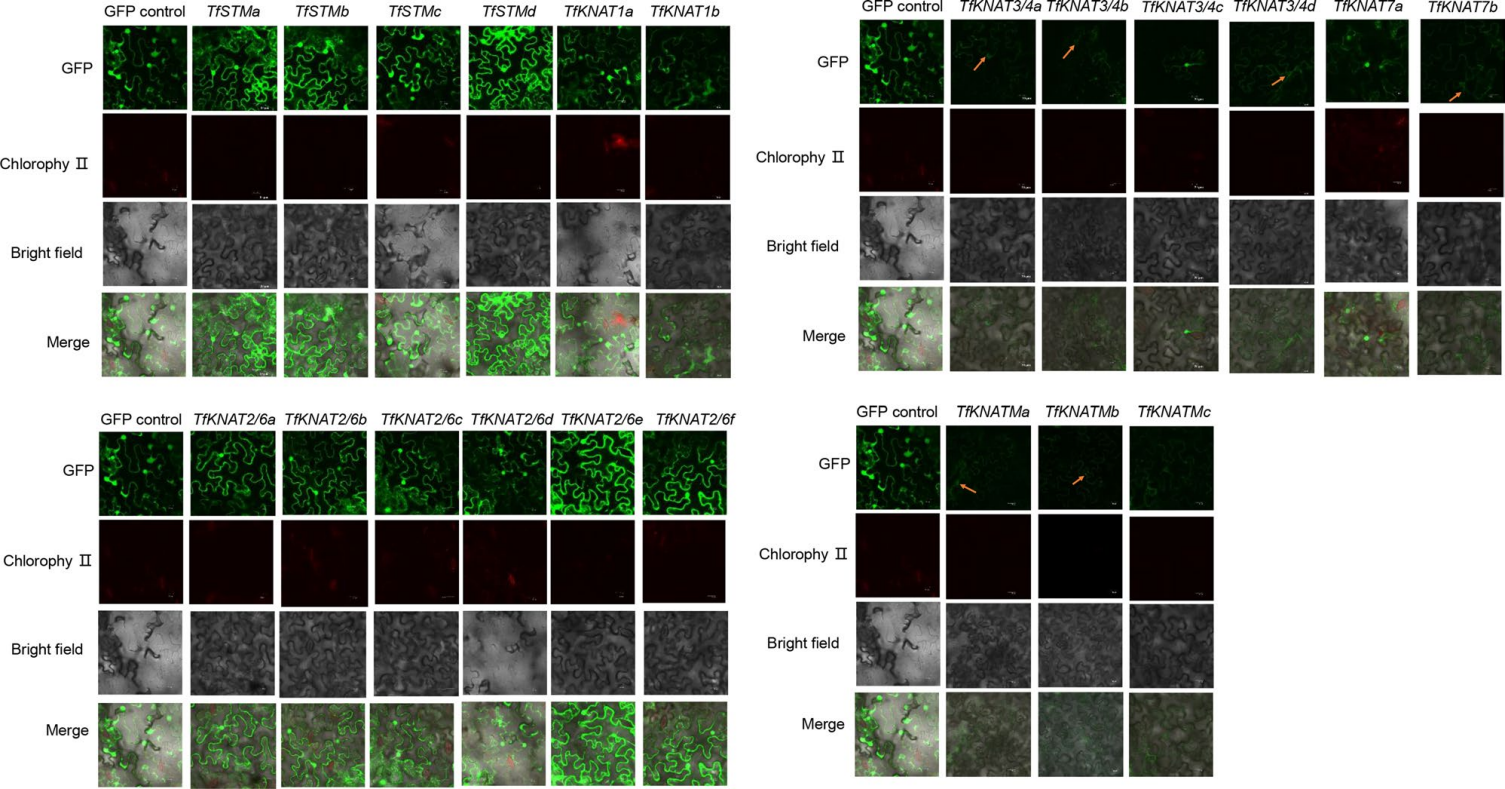
Subcellular location analysis using GFP fused with TfKNATs in leaf epidermal cells of tobacco. The orange arrowheads indicate signals of green fluorescent protein fluorescence

To further verify the subcellular localization of TfKNAT, all the TfKNATs were introduced into the pCAMBIA1304-GFP vector with the CaMV35S promoter. The fusion vectors were subsequently expressed in *N. benthamiana* leaf epidermal cells via *A. tumefaciens*. Most KNOX proteins with green fluorescent protein (GFP) fluorescence, such as TfKNAT1a, TfKNAT1b, TfKNAT2/6a and TfKNAT2/6b, were primarily detected in the nuclei of leaf epidermal cells, and several TfKNATs, such as TfSTMa-d and TfKNAT2/6a-f, were also detected in the plasma membrane and cytoplasm (Fig. 7). These results were partially in accordance with the subcellular localization prediction (Table 1). Generally, class I KNOX genes were localized in both the nucleus and the plasma membrane, and class II KNOX proteins are also primarily localized in the nucleus. The localization signals of class M KNOX members are relatively weak, but they can be also observed in the nucleus.

### Expression profiles of *TfKNAT* genes

**Fig. 8:**
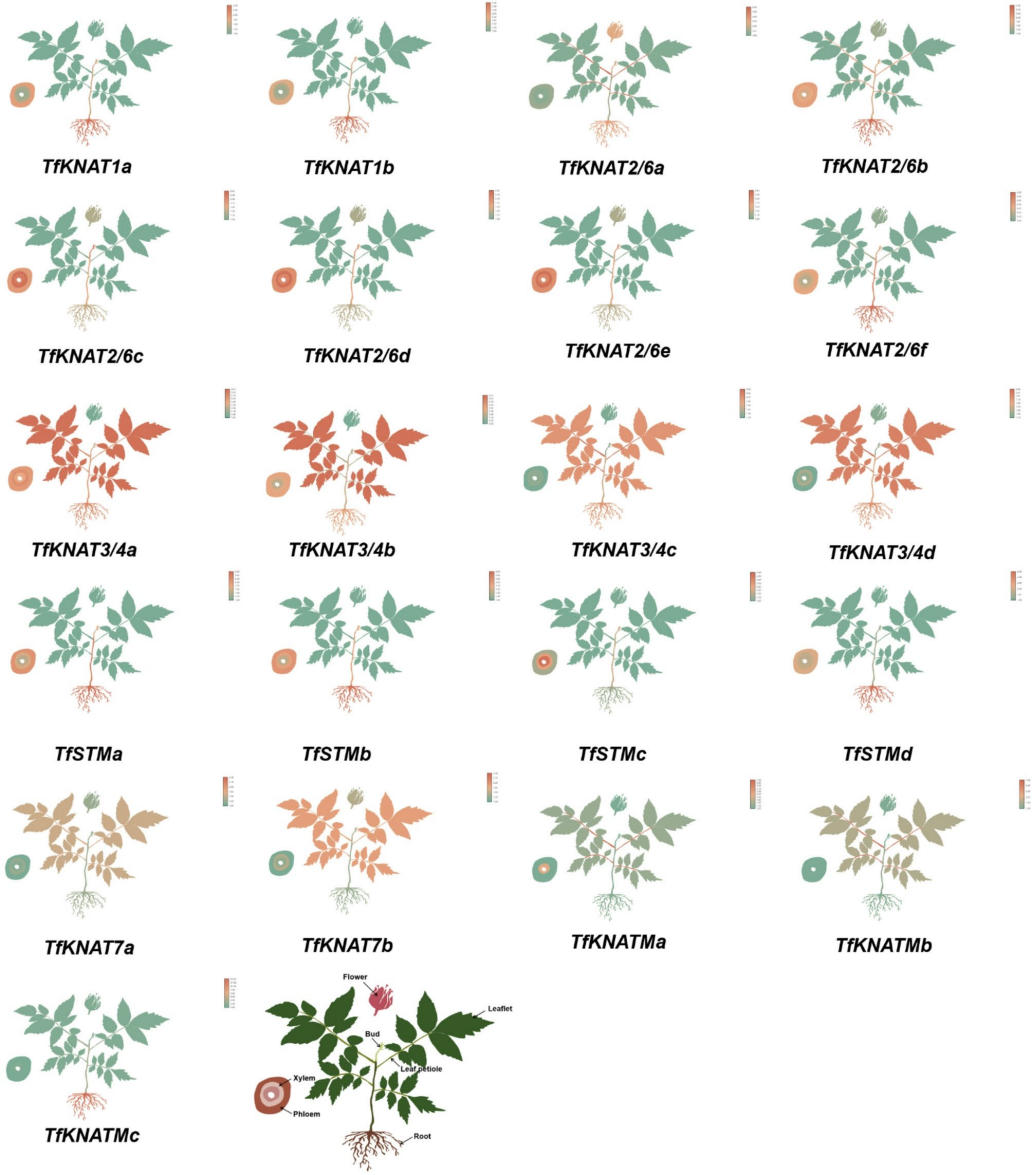
Tissue-specific expression patterns of *KNAT* members. Orange represents high expression of the gene; green indicates low expression of the gene. The last figure illustrates the different tissues of the plant

To clarify tissue-specific expression, comparative analysis of the relative expression levels of *TfKNAT* genes was performed in different tissues, including roots, stems, leaves, buds, petioles, flowers, phloem, primary xylem, and secondary xylem, respectively. To better present the specific expression patterns in different tissues, we sketched the seedlings and different tissues of *T. fargesii* and painted different colors representing gene expression levels (Fig. 8). The class I KNOX genes of *T. fargesii*, with the exception of *TfSTMd*, presented high expression in the leaf buds, which coincided with previous studies reporting that these genes are expressed mainly in the early stage of leaf development. In addition, a few members, including *TfKNAT3/4a*, *TfKNAT3/4b*, *TfKNAT3/4c*, *TfKNAT3/4d* and *TfKNAT7b*, were significantly expressed in the leaflets. *TfKNAT7a* was expressed at low levels in most tissues, whereas *TfKNAT7b* was upregulated in leaflets. Notably, we detected relatively high expression of *TfKNATMa* in roots, but it was seldom expressed in other tissues, indicating its involvement in roots. The gene expression levels varied across different tissues, indicating that family members within the same subclade may have slight divergences. Generally, TfKNAT family members generally play key roles in the growth and development of *T. fargesii*, with each member potentially performing distinct functions across different tissues.

### Expression profile analysis of *TfKNAT* genes under PEG and hormones treatments

**Fig. 9:**
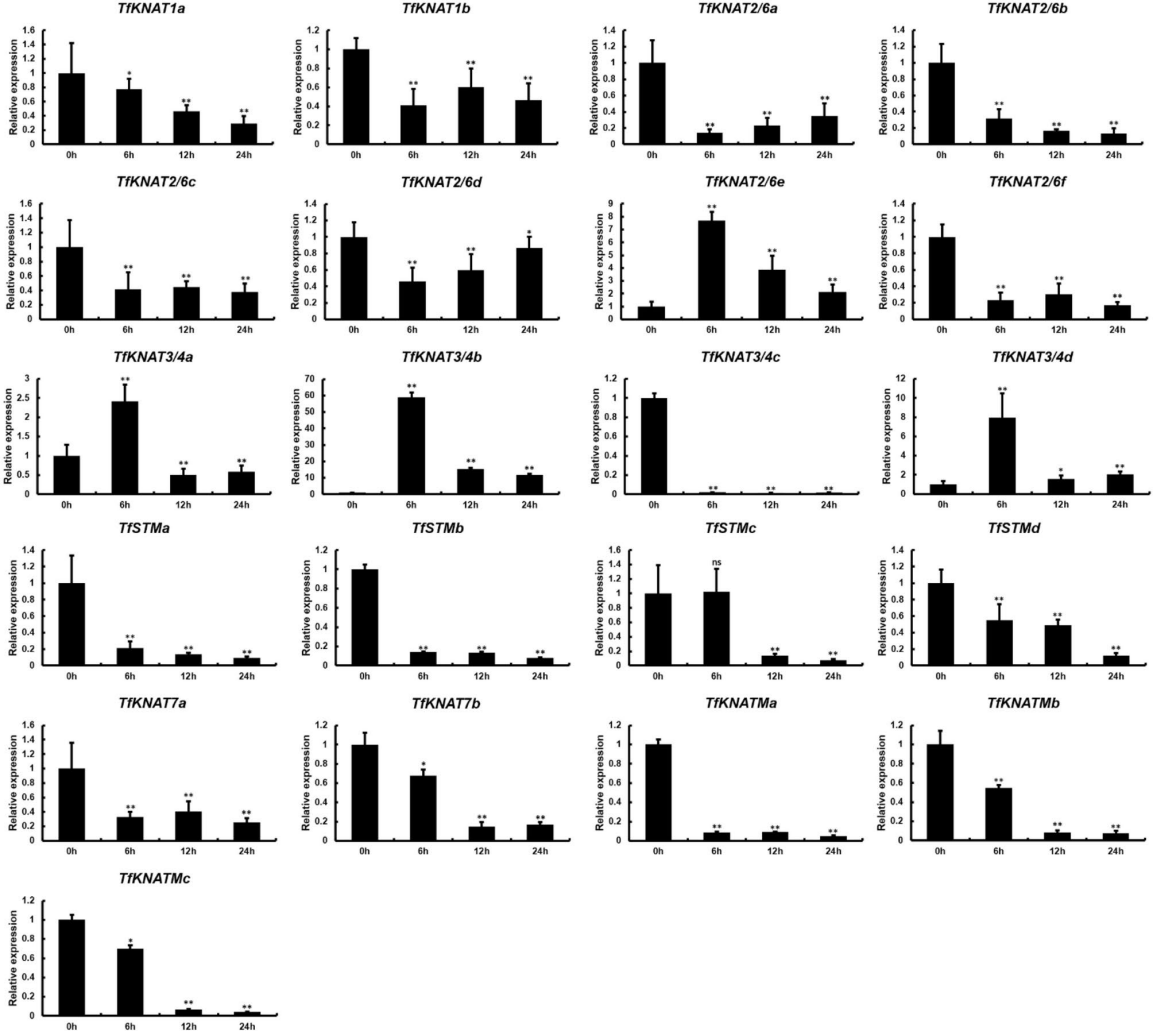
Relative expression of *TfKNAT* genes under PEG stress. Data are presented as mean ± SE at each time point, with *, ** and ns indicating significant differences at *p* < 0.05, *p* < 0.01 and non-significance, respectively

As mentioned above, the *cis*-elements of *TfKNAT* are largely related to drought stress, and water deficiency might be a threat to *T. fargesii*. We therefore detected the expression patterns of *TfKNAT* genes under four durations of PEG stress to further explore the potential function of *TfKNAT* genes with respect to drought stress. The results demonstrated that the relative expression levels of *TfKNAT3/4c*, *TfKNATMa* and *TfSTMa* were significantly declined under PEG stress treatment and tended to decrease with increasing PEG stress duration (Fig. 9), indicating that the stress affected their expression. The expression of *TfKNATMa* and *TfSTMa* was highly expressed in the roots, as presented above (Fig. 8), further suggesting that these genes might be involved in drought stress. Interestingly, the expression of three *TfKNAT3/4* genes including *TfKNAT3/4a*, *TfKNAT3/4b* and *TfKNAT3/4d*, sharply increased and gradually decreased with increasing stress duration. This finding was generally consistent with the analysis of *TfKNAT3/4 cis*-elements, which largely included drought-related elements.

**Fig. 10:**
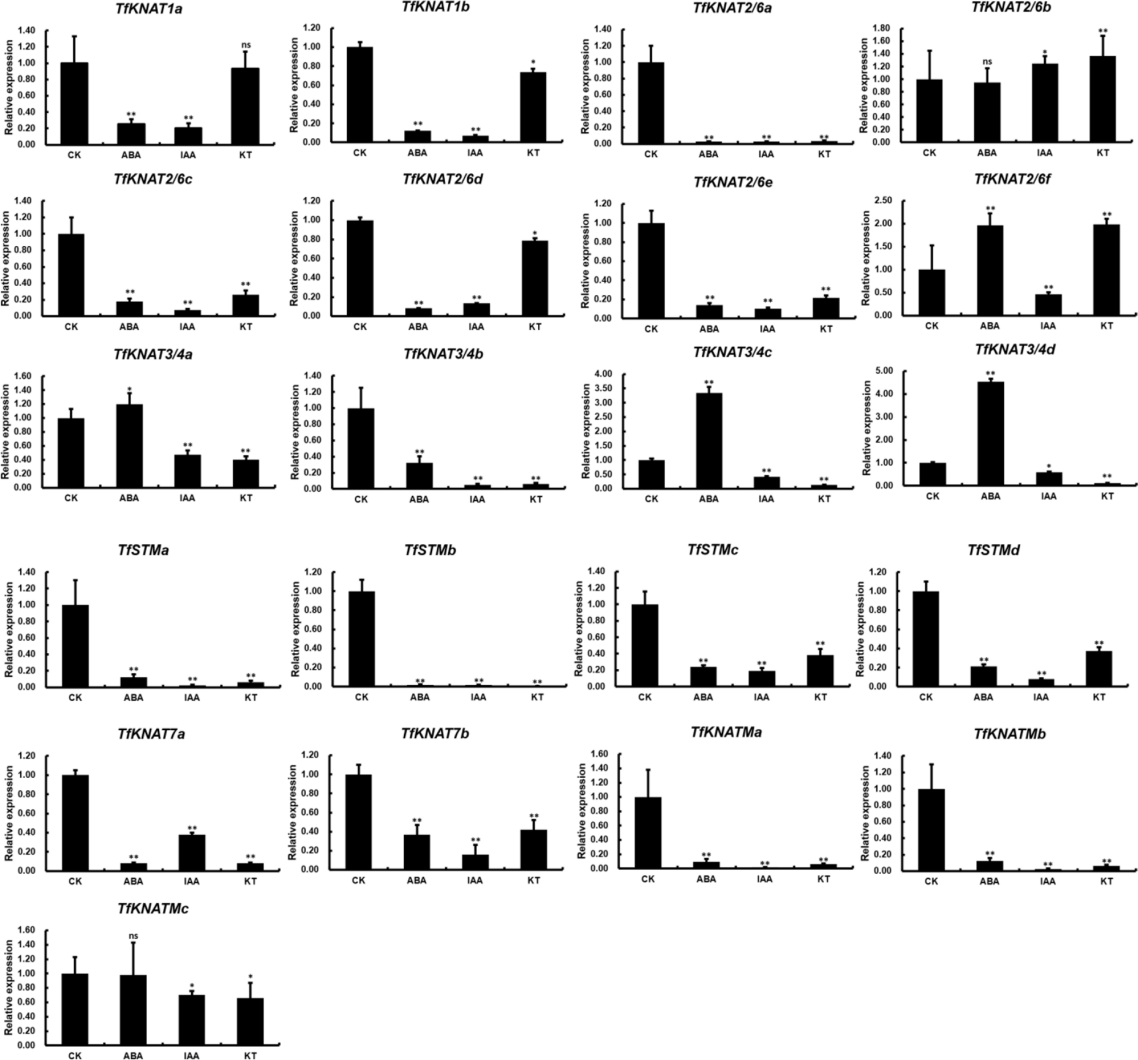
The relative expression of 21 *TfKNAT* genes under ABA, IAA and KT treatments. CK stands for control, ABA for abscisic acid, IAA for indole-3-acetic acid, and KT for kinetin. Data are presented as mean ± SE at each treatment, with *, ** and ns indicating significant differences at *p* < 0.05, *p* < 0.01 and non-significance, respectively

According to previous reports and our above prediction, *KNOX* genes can regulate various plant hormones, particularly auxin, cytokinin and ABA. First, we found that ABA treatment significantly increased the expression of several *TfKNAT* genes. Notably, the treatment enhanced the expression of three*TfKNAT3/4* genes, namely *TfKNAT3/4a*, *TfKNAT3/4c*, and *TfKNAT3/4d*. This result was highly coincidence with previous study and our prediction which indicated that the upstream regions of *TfKNAT3/4* contain many *cis*-elements of ABA response. Additionally, cytokinin treatment led to decrease in several *TfKNAT* genes expression, but most changes were not substantial. Auxin treatment had an inhibitory effect on *TfKNAT* expression (Fig. 10). Generally, we observed that the expression trends of most paralogs showed high similar expression patterns strengthen our hypothesis of KNOX family expansion in *T. fargesii*.

## Discussion

### Identification, classification and evolution of *TfKNAT* genes

Identification and classification are integral for gene family analysis. To better understand the relationships among genes, this study identified *KNOX* genes in *T. fargesii* predominantly by referring to the well-studied *KNAT* genes in Arabidopsis. In Populus (*P. alba × P. glandulosa*), for example, a *KNOX* gene, *pagKNAT2/6b* is named according to its homologs of *KNAT2/6* (KNOTTED-LIKE FROM ARABIDOPSIS THALIANA) in Arabidopsis. Accordingly, the *KNOX* gene in *T. fargesii* was named as *TfKNAT*, reflecting their homologs in Arabidopsis. Notably, among the 21 members, ten pairs of paralogous genes were found in the last branches of the phylogenetic tree, and each pair of paralogs presented the closest phylogenetic relationships, suggesting that the divergence of these paralogs was relatively recent and occurred simultaneously.

*KNOX* genes are versatile regulators and display profound diversity in plant evolution [23, 55]. Previous reviews have summarized the multiple roles played by class I and class II KNOX genes in plant developmental regulation [5, 7, 55, 56]. Class I KNOX genes are expressed mainly in meristems and are involved in transcriptional regulation, whereas class II KNOX genes exhibit more diverse expression patterns and various functions [7]. These genes are generally categorized into three subfamilies (Fig. 1). Regardless of recent duplication of the KNOX family, the class I *KNOX* genes diverged in early seed plants. The first duplication of the ancient *KNOX1* gene likely generated *STM* and another gene, and the second duplication of the latter gave rise to the *BP/KNAT1* and *KNAT2/6* subclades [57]. Here, our results were highly coincidence with these studies. Additionally, a previous study suggested that *KNOX* genes duplicated into two subfamilies after the land plants diverged from algae and before the split between bryophytes and land plants occurred 400 MYA [32, 58]. Since the KNOXM subfamily was defined more recently, it is sometimes overlooked in family analysis. In the phylogenetic tree, we initially omitted the KNATM from Arabidopsis, which is unique in that it lacks the typical HD domain (Fig. 5D). Consequently, we identified a solitary branch among the KNOX members that was unclassified. We then included the members on the solitary branch with the KNATM, and it was successfully classified as the KNOX members under the KNATM subclade (Fig. 1). Additionally, most *TfKNAT* genes possess three typical conserved domains presenting typical KNOX functions. As mentioned above, the study defined a KNOXM subfamily in which the structure of the homeodomain was lost. Similarly, in this study, three members of *TfKNAT* diverged from a distinct group and contained only the two domains KNOX1 and KNOX2, strengthening that they are members of KNOXM (Fig. 5). Indeed, KNATM is in a new subfamily and is conserved in dicotyledons [23], and the KNATM family arose at a later stage during plant evolution, as it is restricted to eudicots [32].

As described in the methods section, BLAST and HMM approaches were both used to identify KNOX family members. The intersection of the results generated by the two approaches identified only 12 genes of *TfKNAT* in *T. fargesii*. Among these, four syntenies were detected and four pairs of orthologs were mapped onto seven pairs of chromosomes. Notably, *TfKNAT7a* and *TfKNATMb* were colocalized on the same chromosome (Chr13), and their paralogs were colocalized on another chromosome (Chr21) with similar locations (Fig. 2A). We speculated that a recent WGD, including at least seven pairs of chromosomal duplications, might have occurred in the species and that many duplications might have been overlooked due to limitations of identification methods. Hence, the KNOX family members were re-identified, and the result selected a combination of BLAST and HMM approaches. A total of 21 *TfKNAT* genes were ultimately obtained through the re-assessment. Ten pairs of paralogs were identified, except for *TfKNATMc*, and strong collinearity were also found between these chromosomes, on which pairs of paralogs were separately localized. For example, Chr7 and Chr11, on which *TfKNAT3/4a* and *TfKNAT3/4b* were separately located, exhibited strong collinearity and similarities in terms of gene density and chromosome length (Fig. 2). Additionally, the 𝐾𝑎/𝐾𝑠 values of the paralogous pairs were both lower than 1.0, and neither tandem duplication nor segmental duplication was found, indicating that the TfKNAT gene family was conserved and underwent purifying selection (Table S4, Fig. 2). The 𝐾𝑠 ranged from 0.15 to 0.25, which is a relatively small range, and we accordingly calculated that the divergence time ranged from 4.2 to 8.4 MYA (Table S4). Interestingly, this time range was close to a reported tetraploidization events (*T. sinensis* special tetraploidization) occurred approximately 15 ~ 17 MYA in *T. sinensis* [59, 60]. Hence, we suggest that TfKNAT family expansion is predominantly driven by the recent WGD events (tetraploidization event) in *T. fargesii*. In summary, the integration of these multiple methods, along with the identification of WGD events, strengthens the reliability of *TfKNAT* identification and provides novel insights into the gene family expansion within the *Toona* genus.

As above illustrated, *TfKNATMc* was the only gene lacking a paralog. To verify our identification, we realigned the *TfKNATMc* sequence with the genome to confirm that no paralogous sequence of *TfKNATMc* exists. On the basis of the above findings, we speculated three possible reasons for this: first, the homologous gene was lost after the WGD (7.9 MYA); second, it was unduplicated during the WGD process; and third, *TfKNATMc* was a novel gene and did not undergo the most recent WGD event. Remarkably, it was reported that the proportion of paralogues among the total collinear genes of *T. sinensis* was 93.60%. However, we speculate that the remaining portion may have been lost due to chromosomal rearrangements, which strengthens the first hypothesis. The specific reasons require further in-depth research.

Furthermore, *TfKNATMc* was highly expressed in roots, highlighting its important roles in root development and adaptation to more recent environmental conditions. With respect to gene expression, since the family analysis revealed that almost all members had a paralog, we designed specific primers for each gene and aligned the primers to the genome sequence to ensure their specificity, thereby avoiding the influence of homologous genes. Interestingly, the expression patterns of most genes were highly similar to those of their paralogs, resembling the co-expression pattern. It also suggests that the WGD event may have contributions to the gene dosage effect. We thus hypothesize that owing to the lack of a homolog, *TfKNATMc* may have crucial functions in roots and compensate for the gene dosage effect by increasing expression levels in roots. Further the functional verification and investigation of *TfKNATMc* may be needed in the future.

### The specific expression pattern of *TfKNAT*

Plants alter their physiology and modify their growth and architecture to adapt to moist environments [61, 62]. As demonstrated above, *T. fargesii* was an endangered species in China owing to issues induced by human beings and poor habitat conditions. Recently, it was reported that *T. ciliata*, a close relative of *T. fargesii*, was likely to continually decline under drying conditions induced by global warming [2]. Indeed, drought stress could be a significant risk to *T. fargesii*, as this species naturally grows near rivers where water is abundant. Hence, we designed a PEG stress experiment and examined *TfKNAT* genes expression. A recent study suggested that *GhKNOX4-A* and *GhKNOX22-D*, two homologs of *KNAT4* and *KNAT3*, contribute to the drought response by ABA coordination ang regulating stomatal opening and oxidative stress in cotton [13]. Similarly, our results showed that the expression of three homologs of *KNAT4*, *TfKNAT3/4a*, *TfKNAT3/4b* and *TfKNAT3/4d*, sharply increased and gradually decreased with increasing drought stress duration, and these *TfKNAT3/4* genes were highly expressed in leaflets. Moreover, the subclade of *TfKNAT3/4* presented the greatest number of ABRE *cis*-elements, which are highly related to ABA indicating that they were sensitive to the drought stress and potentially contributed to drought resistance (Fig. S1). We therefore speculated that most *TfKNAT3/4* genes might be involved in the process of drought stress, which is highly correlated with previous studies [13]. Of these, the ABA treatment was performed to further examine KNOX expression change by the ABA inducing. The expression of *TfKNAT3/4* genes was expectedly upregulated by ABA treatment. This also strengthens our conclusion that *TfKNAT3/4* genes may play crucial roles in drought response through ABA coordination. Notably, the PEG treatment used in this study simulates drought stress rather than applying it directly for two main reasons. First, drought stress treatment may involve the combined effects of various environmental factors such as temperature, air humidity and soil moisture content, whereas PEG treatment provides a more controlled simulation of drought stress. Second, the use of PEG treatment is based on relevant studies from previous studies [50, 51]. Furthermore, the purpose of this work is to analyze the characteristics of the KNOX family in *T. fargesii*, so the PEG treatment is more controllable, which may better facilitate our objectives. The further information between the identified *TfKNAT3/4* genes and drought stress requires further investigation. In this study, the *cis*-regulatory elements of class II *KNOX* genes were highly diverse and that class II *KNOX* gene was extensively expressed in different tissues (Fig. 9). These implied that class II *KNOX* genes might be involved in multiple biological processes and highlight the evolutionary significance of class II KNOX genes in *T. fargesii*. In addition, transgenic plants in which *STM* is overexpressed are more tolerant to drought stress, suggesting that *STM* contributes to drought resistance in Arabidopsis [11]. Our results showed that the *TfSTMa and TfSTMb* genes presented relatively high expression in roots but a decreasing trend in gene expression under drought stress. Conversely, its ortholog, *TfSTMc*, tended to increase and then decrease in gene expression with increasing drought stress duration but exhibited low expression in roots, indicating that *TfSTMc* might involve in drought stress and there could be slight divergence among the members of *TfSTM* in *T. fargesii*. Therefore, we would like to know if *TfKNAT* genes were tissue-specifically diverged and therefore carried out further tissue-specific expression pattern analysis. The tissue-specific expression analysis further validated our hypothesis that was most paralogous genes displayed similar tissue-specific expression patterns.

All the species in the *Toona* genus are deciduous trees with pinnate compound leaves, such as *T. fargesii*, *T. ciliata* and *T. sinensis*, and they are ideal materials for exploring compound leaf formation. As vital regulators that greatly contribute to the complexity of leaves, *KNOX* genes, especially class I *KNOX* genes, are well known for promoting compound leaves. A previous study elucidated that the evolution of *A. thaliana* involved a recent selective sweep, *cis*-regulatory divergence, which involved the loss of *STM* leaf expression in *A. thaliana*, resulting in unlobed leaves [63]. Recently, a study revealed that the class II KNOX family is also involved in controlling compound leaf patterning in *M. truncatula* [36]. In this work, all the class II members, *TfKNAT3/4a*, *TfKNAT3/4b*, *TfKNAT3/4c*,* TfKNAT3/4d* and *TfKNAT7a* exhibited high expression in the leaflets of compound leaves, suggesting that their putative functions in compound leaf formation. These results might provide genetic information on compound leaf formation and evolution in the *Toona* genus. Additionally, *T. fargesii* is also an economic wood tree whose timber is used for manufacturing high-grade furniture and decorations. Notably, KNOX genes serve as negative or positive regulators involved in several processes of wood formation. For instance, class II *KNOX* can repress cell wall thickness during wood development in many plants [34, 36, 64, 65]. As mentioned above, overexpressing *PagKNAT2/6b* resulted in altered vascular patterns characterized by decreased secondary xylem with thin cell walls containing less cellulose, xylose and lignin in poplars. In this study, *TfKNAT2a* and most of class II *KNOX* including *TfKNAT3/4c*,* TfKNAT3/4d*, *TfKNAT7a* and *TfKNAT7b* were significantly downregulated in the xylem within the stem, indicating their similar functions with respect to wood quality. However, heartwood formation, by no means, solely coordinates through *KNOX* genes, and it is worth verification in the future. Even if we examined these paralogous genes, the divergences among these paralogs also underlie the emergence of novel functions or functional divergences.

### Future studies on TfKNAT and gene evolution in Meliaceae family

Although many studies have conducted genome-wide identification of gene families in several species of the *Toona* genus, this study first time reports a significant duplication induced by WGD within a gene family. Given that paralogous genes present high sequence similarity and that their expression patterns have not largely diverged, several factors need to be considered in molecular biology experiments. For example, the karyotype of *T. fargesii* partially resembles a putative tetraploid due to the WGD but the paralogous pairs have not totally diverged in their expression patterns. Similar cases may also exist in other species of the *Toona* genus, including *T. sinensis* and *T. ciliata*. Even though no significant divergence between the paralogous pairs has been found in the sequences, gene structures, collinearity relationships, and expression patterns of these genes, more evidence is needed to determine whether they have diverged functionally. Besides, only the TfKNAT family exhibited the apparent duplications in this study, the other families may need to be further identified in the future in the *Toona* genus.

The species of the *Toona* genus always contains 28 pairs of chromosomes, such as *T. fargesii*, *T. sinensis* and *T. ciliata* [59], but the species of the *Melia* genus usually has 14 pairs of chromosomes, which is half as many. Whether strong synteny exists between the *Toona* genus and the *Melia* genus also needs further research for revealing the details of the evolution of the Meliaceae family. Although a genome paper of species in *Melia* genus, such as *M. indica*, has been published, the genomic data have been unreleased yet [60]. It can only be expected that more genomic data will be released in the future, which is expected to provide further supporting evidence.

## Conclusion

In this study, we identified a total of 21 members of the KNOX family in the *T. fargesii* genome using multiple methods. Generally, *TfKNAT* genes were classified into three subfamilies: class I, class II, and class M, based on phylogenetic relationships. Notably, ten paralogous pairs were detected, each exhibiting high similarity in sequences, gene structures, *cis*-elements, subcellular localizations and expression patterns. Evolutionary analysis indicated that a specific WGD event greatly drove the expansion of the TfKNAT family. These results suggest that the duplicated genes have not significantly diverged from their original counterparts, with only slight differences observed between paralogous pairs. Additionally, the *cis*-regulatory elements of class II KNOX genes are more diverse than those of the other two subfamilies, partially explaining the extensive expression patterns of class II KNOX genes across different tissues. Furthermore, three *TfKNAT3/4* genes contained a large number of *cis*-regulatory elements related to drought stress, and their expression levels sharply increased and then gradually decreased with prolonged PEG stress, indicating their role in drought stress response. Moreover, ABA treatment induced three *TfKNAT3/4* genes expression indicating that these genes may play significant roles in drought response through ABA hormone mediation. Collectively, this study highlights the KNOX family genes characterization, evolution and expression and indicates *TfKNAT3/4* genes may contribute to the drought resistance in *T. fargesii.*

## Electronic supplementary material

Below is the link to the electronic supplementary material.

Supplementary Material 1

Supplementary Material 2: Table S1 The primer sequences for qRT-PCR experiment

Supplementary Material 3: Table S2 The primer sequences for the PCR experiment

Supplementary Material 4: Table S3 Protein sequence of KNOXs from *T. fargesii*, *Arabidopsis thaliana*, *Oryza sativa*, *Solanum lycopersicum*, *Zea mays*, *Allium sativum* and *Populus trichocarpa*

Supplementary Material 5: Table S4 The divergence estimation of the paraplogs

## Data Availability

The genome raw reads produced in this study have been deposited in the CNCB genome sequence archive (GSA) under accession number GWHEQVK00000000 (https://ngdc.cncb.ac.cn/).
