# Supplementary material for: Genome-wide identification and integrative analysis of KNOX family characterization, duplication and expression provide insights into PEG-induced drought stress in Toona fargesii
Source: BMC Genomics. 2025 Apr 29;26:423. doi: 10.1186/s12864-025-11628-4 (PMC12038949; doi:10.1186/s12864-025-11628-4)
Supplement: Supplementary file 1 — Supplementary Material 1 [file 12864_2025_11628_MOESM1_ESM.docx]

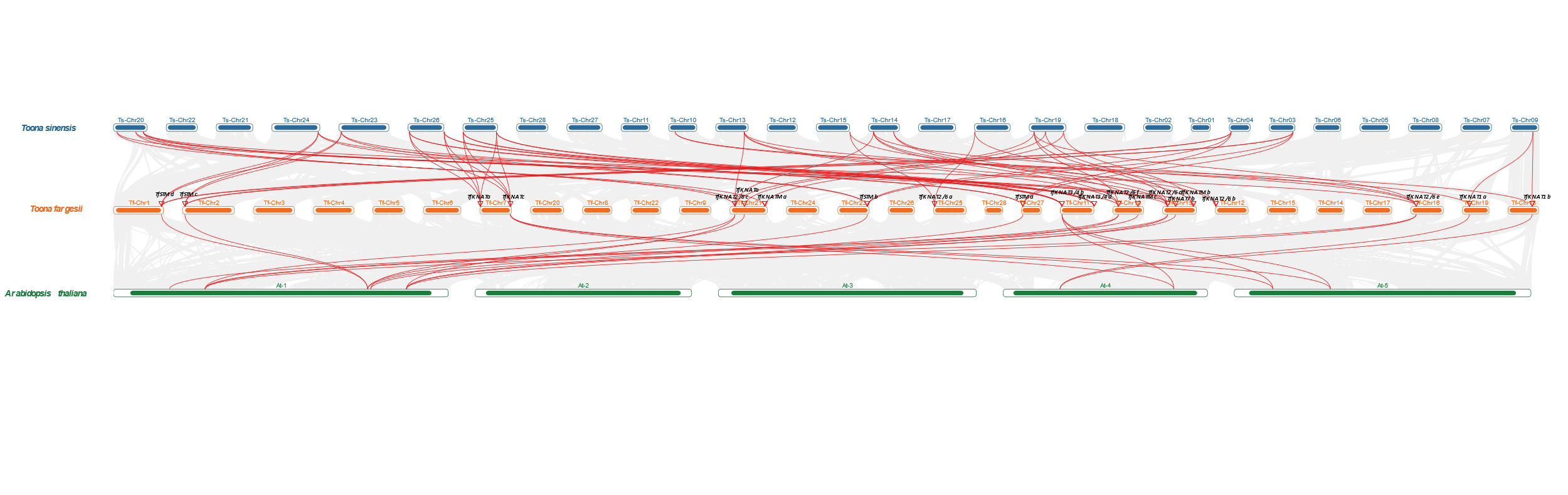


**Fig. S1** Analysis of gene synteny between *Toona fargesii* and *Toona sinensis*, and the synteny between T*oona fargesii* and *Arabidopsis thaliana*. The red lines indicate *KNOX* genes.


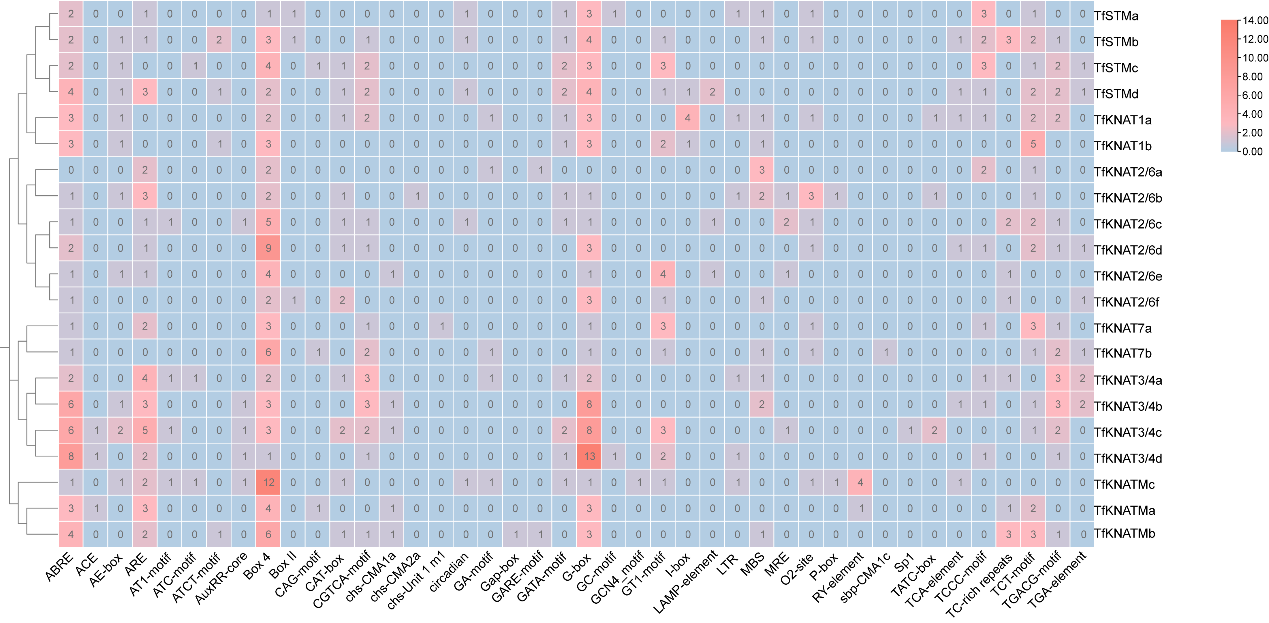


**Fig. S2** The *cis*-elements numbers of TfKNAT.
